# Frequently Consumed Foods and Energy Contributions among Food Secure and Insecure U.S. Children and Adolescents

**DOI:** 10.3390/nu12020304

**Published:** 2020-01-23

**Authors:** Heather A. Eicher-Miller, Carol J. Boushey, Regan L. Bailey, Yoon Jung Yang

**Affiliations:** 1 Department of Nutrition Science, Purdue University, 700 W State St, West Lafayette, IN 47907, USA; cjboushey@cc.hawaii.edu (C.J.B.); reganbailey@purdue.edu (R.L.B.); yang3@purdue.edu (Y.J.Y.); 2 Epidemiology Program, University of Hawaii Cancer Center, 701 Ilalo St, Honolulu, HI 96813, USA; 3 Department of Food and Nutrition, Dongduk Women’s University, 60 Hwarang-ro 13-gil, Wolgok 2(i)-dong, Seongbuk-gu, Seoul 136714, Korea

**Keywords:** food group intake, child food security, popularly consumed foods, low-resource children, adolescents, food intake, beverage intake, dietary intake, food insecurity, US children

## Abstract

Food insecurity is associated with nutritional risk in children. This study identified and compared the most frequently consumed foods, beverages, and food groups and their contributions to energy intake among U.S. children and adolescents (6–11, 12–17 years) by food security status. Dietary intake from the day-1, 24-h dietary recall, and household child food security status were analyzed in the 2007–2014 National Health and Nutrition Examination Survey (*n* = 8123). Foods and beverages were classified into food categories, ranked, and compared by weighted proportional frequency and energy contribution for food security groups by age. Significant differences between household child food security groups were determined using the Rao-Scott modified chi-square statistic. The weighted proportional frequency of beverages (including diet, sweetened, juice, coffee, and tea) and their energy was significantly higher among food insecure compared with food secure while the reverse was true for water frequency among 12–17 years. Beverage and mixed dish frequency were higher among food insecure compared with food secure 6–11 years while the reverse was true for frequency and energy from snacks. Frequency-differentiated intake patterns for beverages and snacks by food security across age groups may inform dietary recommendations, population-specific dietary assessment tools, interventions, and policy for food insecure children.

## 1. Introduction

The U.S. Dietary Guidelines for Americans Advisory Committee identified many children and adolescents as having low intakes of fruits, vegetables, whole grains, and dairy concomitant with excessive intakes of sodium, saturated fats, added sugars, and refined grains [1]. Such dietary patterns are linked with nutritional risk, or dietary deficiencies that endanger health, as age progresses through childhood. Low micronutrient intakes combined with excessive energy intakes culminate in adolescence, when growth is accelerated and nutrients are at highest demand and yet this age group has the most nutrient shortfalls across the lifespan [2].

Adolescents and children in food insecure households, with “limited or uncertain availability of nutritionally adequate and safe foods or limited or uncertain ability to acquire acceptable foods in socially acceptable ways” [3], may be particularly vulnerable to nutrition risk, increasing the likelihood of suboptimal cognitive and physical health [4,5,6]. Indeed, iron deficiency anemia and low bone mineral content were associated with food insecurity in childhood as were behavioral and mental health problems, and poorer general health [7,8,9,10]. These associations may stem from disparities in dietary intake among food insecure children [11] where the opportunity for divergence from recommended dietary patterns is high considering limited household budget, time, and other resources. For example, a recent systematic review among U.S. children found strong and consistent evidence of higher added sugar intake among food insecure children 6–11 years compared to those who were food secure [11]. Food insecurity is particularly salient in the U.S. as 3.1 million or 8% of households with children in 2016 were food insecure: 7% low food security or “reduced quality and food access problems” and 1% very low food security or “reduced food intake and disrupted eating patterns” because of inadequate food resources [12].

However, little is known about the specific eating patterns and food and beverage exposure patterns among U.S. children and adolescents with regard to food security status. Eating patterns, including frequency and amount of foods and beverages consumed, snacking and meal skipping, time of eating occasions and other eating behaviors, influence energy intake and contribute to dietary quality [13]. Research on these patterns was a data gap in the Scientific Report of the 2015 Dietary Guidelines Advisory Committee along with investigation of foods comprising the U.S. food environments, particularly for food insecure households and low-income individuals [1]. Knowledge of the specific frequently consumed foods is a novel and practical contribution to inform interventions and policies aimed to improve dietary quality and food security among children. For example, results may inform a food package of nutrient-dense foods already known to be familiar and often consumed among food insecure children. Therefore, the purposes of this study were to use the National Health and Nutrition Examination Survey (NHANES) 2007–2014 data to: (1) determine the foods and beverages and categories of foods and beverages most frequently consumed by food security status (food secure, low food secure, and very low food secure) in children (6–11 years) and adolescents (12–17 years), and (2) compare the energy contributions and frequency of reported intake of food and beverage categories by food security status.

## 2. Materials and Methods

### 2.1. NHANES Design

NHANES is a nationally representative, cross-sectional survey of the National Center for Health Statistics (NCHS) and Centers for Disease Control and Prevention [14,15]. The non-institutionalized, civilian U.S. population are sampled based on characteristics such as age, sex, race-ethnicity, and income to accommodate the complex, stratified, multistage probability sampling framework [16]. Oversampling of certain sub-groups allows for generation of reliable estimates. NHANES protocol was reviewed and approved by the NCHS Research Ethics Review Board [17].

### 2.2. Participants

All participants of this secondary analysis completed the dietary component of What We Eat in America (WWEIA)/NHANES 2007–2008, 2009–2010, 2011–2012, and 2013–2014. Children were 6–17 years (*n* = 8,123, Table 1), having a 24-h dietary recall, dietary weights and scores for the U.S. Household Food Security Survey Module [18]. Socioeconomic characteristics of participants were recorded in participant homes during an in-depth interview for those 16–17 years and a proxy-assisted interview for those 6–15 years. Age (6–11 or 12–17 years), gender (male or female), survey year (2007–2008, 2009–2010, 2011–2012, 2013–2014), poverty-income-ratio (0.00–0.99, 1.00–1.99, 2.00–2.99, 3.00–5.00), race/ethnicity (non-Hispanic white, non-Hispanic black, Hispanic and Mexican American, and “other” race including multi-race), and weight status as indicated by body mass index (underweight, normal weight, overweight), characterized participants. Per NCHS analytic guidelines, “other” race is not representative of race/ethnic population estimates.

### 2.3. Measures

One adult per household completed the 18-item U.S. Household Food Security Survey Module for households with children <18 years during the household interview. Eight child-focused items determined food security of household children and were used to classify food security, low and very low food security; low and very low categories were also collapsed to classify food insecurity [18]. Food security of household children rather than the entire household was chosen as more directly tied to the child experience and dietary intake of household children. Measures of height and weight were collected during a physical examination at the Mobile Examination Center. Body mass index was calculated as body weight divided by the square of body height and categorized according to age- and sex-specific percentiles of the 2000 Centers for Disease Control and Prevention growth chart such that <5% (underweight), 5 ≥ 85% (normal weight), 85 ≥ 95% (overweight), ≥95% (obese) to indicate weight status [19].

The day-1 dietary recall was completed in person at the Mobile Examination Center using the USDA Automated Multiple Pass Method, designed to enhance food recalls using a 5-step interview process [20,21]. Participants were prompted to recall all types and amounts of foods and beverages (including water) consumed in the 24-h midnight to midnight time frame before the interview. Children 6–11 years reported dietary intake with the assistance of a parent or guardian, those 12–17 years self-reported. Probes queried the time and eating occasion of foods, details about preparation and amounts eaten, and finally, any frequently forgotten foods and foods not mentioned earlier. A USDA food code was assigned to each reported item and linked to a food or beverage in the Food and Nutrient Database for Dietary Studies (version 4.1 released 2010, 5.0 released 2012, 6.0 released 2014, 2013–2014 released 2016) [22], and further sorted and assigned a WWEIA food sub-category/group and broad food category/group [14].

### 2.4. Statistical Analysis

The data of food secure and food insecure children, including low and very low food secure categories were stratified by ages: 6–11 and 12–17 years because of similar diets within age ranges, food security reporting, known differences in food security by age in the same household and the NHANES methodology of self-reported dietary recall by age groups. Despite small participant *n* for the very low food secure group, hypothesis testing was included because food category reports were the unit of analysis and *n* >20 for all food categories except “alcohol” and “other” including infant and baby formula (excluded from Table 2). Food category reports of “water” contributing energy were also <20 but were retained for comparison with frequency. Unadjusted frequencies were assessed for each food code or WWEIA food or beverage category code using: *n*∑i = 1(Ri) where *n* = the sample size, i = each participant, Ri = the number of reports of individual food codes for the ith individual [23]. The weighted sum of each food code was: *n*∑i = 1(Riwi) where wi = sample weight for the ith individual was used to determine the weighted proportion of foods to the total foods reported, or the contribution of each food category reported to the total food category reported, given as: *n*∑i = 1(Riwi)/*n*∑i = 1(Tiwi) (100) where Ti = total number of reports of all food codes for the ith individual. The weighted proportion of reported energy was similarly calculated with substitution of energy for frequency and total energy for total number of reports. Foods were ranked by weighted frequency and contribution to energy individually, by food sub-category and broad category (selected data shown in tables). The Rao-Scott modified chi-square determined significant differences among food secure, low, and very low food secure groups (*p* < 0.05/3 or *p* < 0.02 using a Bonferroni adjustment for multiple comparisons to mitigate the probability of Type 1 error) and among food secure and insecure groups (*p* < 0.05). The results of significant differences among broad food groups were used to focus presentation of the results and discussion. All analyses were completed in SAS version 9.4 using SAS survey procedures with adjustment for survey design elements, non-response, and interview weights to allow inference to U.S. population.

## 3. Results

Overall, ~90% of U.S. children and adolescents were food secure and 10% food insecure, with the smallest proportion being very low food secure (1–2%). Household poverty-income-ratio and race/ethnicity differed among 6–11 and 12–17 years by food security status (*p* ≤ 0.0004, Table 1) as did the prevalence of at-risk-for-overweight and overweight only among children 6–11 years (*p* = 0.001).

### 3.1. Frequency and Energy Contribution of Broad Food Categories, Sub-Categories, and Foods

The broad food categories, energy contributions and reported frequency of consumption, were compared by food security status for ages 6–11 and 12–17 years in Table 2. Broad food category rankings by frequency and energy contributions were also considered. Ranking revealed broad category “snacks and sweets” as the most frequently consumed items for all children 6–11 years (Table 2). Broad category “beverages” were second or third most frequently consumed but ranked sixth in terms of group contributing to energy. Among those 12–17 years, “snacks and sweets” shared the top ranking with “beverages” and where ranking differed by food security status. “Beverage” contribution to energy ranked third to fifth. “Mixed dishes” ranked lower in frequency compared with contribution to energy ranking among both age groups and all food security categories. “Milk and dairy”, “grains”, and “protein foods” also had high rankings in both frequency and energy contribution for all ages and food security categories. “Water” and “condiments” added little to energy but ranked higher in terms of frequency.

#### 3.1.1. 6–11 Years

The weighted proportion of the broad category “beverages” (*p* = 0.02, Table 2) and “mixed dishes” (*p* = 0.04) reported by frequency was statistically significantly greater for food insecure compared with secure children 6–11 years (12.7% vs. 11.4%, Table 2). “Mixed dishes” were also more frequently reported among food insecure at 9.3% compared with food secure at 8.0%. In contrast, reported intake of “snacks and sweets” by frequency (*p* = 0.02) and energy contribution (*p* = 0.02) was lower among food insecure compared with secure children of similar age (14.4% vs. 16.1% and 18.3% vs. 21.0%). Additional significant differences resulted among food secure, low and very low food secure groups (*p* = 0.02) for “snacks and sweets” (21.0%, 18.1%, 20.1%).

Food sub-categories contributing to the broad beverage category such as “fruit drinks” captured 3.2%, 4.3%, and 4.1% of reports (Table 3) among food secure, low, and very low food secure children. The pattern was consistent with lower “soft drink” reports for food secure (3.0%) compared with low (3.9%) and very low (3.4%) food secure children. Top items in these sub-categories were “fruit flavored drink from powder”, “fruit-flavored caffeine-free soft drink”, “cola-type soft drink”, “apple juice”, “orange juice”, “fruit juice drink”, and “reduced sugar fruit juice drink” (Supplemental Table S1). The broad “snacks and sweets” category included sub-categories, “cookies and brownies” and “candy without chocolate”, with a higher percentage of reports among food secure (both 2.6%) compared with low (1.9%, 1.6%, respectively) and very low (1.5%, 2.2%, respectively) food secure children. “Corn tortilla chips”, “hard candy”, “chocolate chip cookie”, “ice cream” and “snack crackers” were most frequently consumed items in these sub-categories.

#### 3.1.2. 12–17 Years

Compared with food secure adolescents 12–17 years (12.7%) “beverages” as a broad category were more statistically significantly frequently consumed by low (15.5%) and very low food secure (14.4%) and also combined food insecure groups (15.4%, *p* = 0.0001). A greater contribution of energy from “beverages” (*p* = 0.03) was also determined for food insecure compared with secure (13.0% vs. 11.2%). Alternatively, significantly more frequent intake of “water” was observed among food secure (*p* = 0.004), compared with insecure and low and very low food secure groups (10.3%, 8.7%, 8.6%, 8.8%).

Food secure adolescents reported 4.3% intake frequency of “soft drinks” contrasting with the similar pattern of higher intakes for low food secure at 5.6%, the most frequently consumed sub-category for this group, and 5.1% for very low food secure adolescents as in the younger age group, but with an even greater percentage of reports. Top items were “cola-type soft drink”, “fruit-flavored, caffeine free soft drink”, “brewed sugar-sweetened iced tea”, “orange juice”, “fruit flavored drink from powder”, “fruit flavored soft drink”, “fruit juice drink”, and “apple juice”. “Tap water” had the reverse pattern as “soft drinks” and comprised the most (5.9%) reports among food secure adolescents while accounting for 4.5% and 3.5% of low and very low food secure reports. “Bottled water”, however, was less frequently consumed among food secure and low food secure groups (4.0%, 4.1% respectively) compared with very low (5.2%) food secure reports. “Tap water” and “unsweetened bottled water” were the most frequently consumed items.

## 4. Discussion

Both the frequency and amount of food and beverage intake are important behavioral exposures characterizing dietary intake. Frequency data permits consideration of the most commonly consumed foods while amount shows the “dose”. In this analysis, U.S. children and adolescents had similar frequency of consumption of food categories regardless of food security with the exception of beverage and snack categories. Frequency alone is often overlooked as a component of dietary patterns among children and only two studies are known among adults [23,24]. Traditional dietary assessment, namely food frequency questionnaires, have relied on querying frequency to obtain results focused on contributions to servings of foods, energy and nutrients. Yet, separation of frequency from energy contribution and consideration of frequency as a dietary behavior with potential links to health presents opportunities for behavioral interventions. “Beverages” or “snacks and sweets” were the most frequently consumed broad food groups for all children 6–17 years yet neither ranked as highest contributor to energy, exemplifying their potentially under-recognized importance in children’s diets. Their ubiquitous frequency represents potentially impactful targets for intervention to improve overall dietary quality to develop healthy habits for later life [25]. “Beverages” as a broad category represents a spectrum of product types, some without and others with added sugars and key nutrients, respectively [1,14]. However, sub-categories and individual foods ranked by frequency reveal that beverages with a high amount of added sugars are prominent choices [26]. Thus, particular attention to intervention messaging and counseling to improve drink choice among children should be provided. Recommendations for specific beverages and not only broad categories, may be gleaned from these frequency rankings and used to educate healthful patterns. High frequency of snacks and sweets, particularly candy, cookies and brownies, and ice cream, may be targets of interventions more clearly interpreted to broadly limit because of their inherent added sugars [26], yet perhaps more difficult, compared with beverages, to find acceptable substitutions.

Frequency differences in “beverages” and “snacks and sweets” by food security status supports a previous summary of the literature for children 6–11 years for higher added sugar intake [11], sourced from beverages, snacks and sweets among U.S. children [26], and are novel among the sparse evaluation for those 12–17 years [11,27]. “Beverage” intake frequency associated with food insecurity in both age groups may potentially be a manifestation of choices prioritized to satisfy hunger rather than health. Less frequent “water” intake among food insecure adolescents 12–17 years may be related, as a trade-off for higher intake of other beverages [28] or due to lack of potable water supply access among food insecure households [29]. These results support findings showing higher odds of heavy (i.e., more energy) total sweetened beverage intake among low-income compared with high-income children 2–11 years [30]. Older children may be making independent dietary choices and may also be encouraged to obtain food outside the household when also food insecure.

“Soft drinks” were the highest ranking items in the “beverages” category among older food insecure children with caffeinated soft drinks ranking prominently. Older, prevalently low-income children may be working and contributing to family income [31] and using caffeinated beverages to maintain their schedules [32]. Intake frequency and timing of caffeinated beverages may matter more to healthful sleep/wake habits compared with total intake. Previous observational studies have suggested that consumption of caffeinated beverages leads to sleep dysfunction in junior high and high school children [33], and associate with obesity among children 11 years of age [34]. High frequency of caffeinated soft drinks is consistent with these observational results and offers additional evidence supporting dietary interventions to reduce caffeine intake and frequency among all children and especially older food insecure children.

Less frequency and energy contributions of “snacks and sweets” among food insecure compared with secure may represent a relatively more healthful dietary pattern among food insecure 6–11-year-old children. “Snacks and sweets” may be viewed as non-essential foods where budget may be conserved and include high-energy, high-sodium foods [25,35]. As such, the results are unaligned with previous explanations of dietary differences among food insecure groups generalizing a reliance on high energy, low nutrient foods [36].

Frequently consumed foods and beverages among children and adolescents may be used to inform opportunities to promote available and familiar foods that are sources of the nutrients or dietary components that are lacking [11,27], and inform efforts to build on dietary strengths by promoting foods that are already frequently consumed. For example, cow’s milk and raw apple were highly reported and may be further promoted in children’s food environments. The prominence of “condiments” among frequently consumed foods was apparent. Items like catsup, mustard, mayonnaise, and salsa consumed in relatively small amounts and with little contribution to energy may be used to enhance taste. Their frequent use presents an opportunity to for stealth nutrition interventions to potentially fortify condiments with nutrients most children need more of, and to further reduce components most children need less of (e.g., sodium and sugar).

### 4.1. Implications

While applications of stealth nutrition may help, the overall poor dietary intake of U.S. children regardless of food security status demand more dramatic changes to improve dietary selection. Primary care contact or public health education among youth provide an ideal environment for education and discussion of dietary habits and suggestions for substitution of soft drinks, for example, with water or low-fat dairy and promotion of fruits and vegetables. Dietary recommendations for food groups and categories may be further translated to specify frequently consumed foods comprising groups recommended for increase or decrease. Federal nutrition assistance programs such as Supplemental Nutrition Assistance Program; Special Supplemental Nutrition Assistance Program for Women, Infants, and Children; and the National School Lunch and Breakfast Programs may similarly apply knowledge of the frequently consumed foods to tailor education and menu components to the 59% of 2016 U.S. food insecure households participating [12]. The National School Lunch and Breakfast Programs play key roles in child nutrition as they represent two main eating occasions of a child’s day. Vegetables and fruits may be further promoted through these programs in order to increase their frequency and contributions to total energy. Frequently consumed foods may be key foods for companies to consider nutrient profile improvement to reduce added sugars, sodium and increase calcium, vitamin D, potassium, and fiber [1]. Examination of frequently consumed foods by age group can inform dietary intake questionnaires and be used to populate technology-assisted dietary assessment search tools. Finally, foods listed by frequency may inform monitoring of population dietary intake and potential food environment improvements that enhance safe access to enough foods for healthy, active lifestyles [37].

### 4.2. Strengths and Limitations

Dietary intake and reporting are reliant on memory and prone to error [38]. Much less is known about the measurement error in children and adolescents self-reported diets compared with adults. Dietary recalls throughout the week allow representation of week and weekend days for U.S. children contributing a strength, yet this analysis is limited as it only represents one day of data for each participant and does not reflect usual intake over time. Aggregation to broad and sub-food categories may highlight food group differences that depend on the groups combined while dis-aggregation may highlight differences that are not meaningful to nutrition such as ‘tap water” vs. “bottled water”, yet use of broad and sub-food categories aligns with the practical translation of dietary recommendations. Lastly, since food security among household children is reported by a household adult, an individual child’s food security may be biased by the perception of the adult and the adult’s perception of food security for other children in the household [39]. Older children tend to be under-classified as food insecure while younger children may be over-classified. While imperfect, differences by food security and age were observed in this analysis and add knowledge of the dietary patterns of food insecure children.

## 5. Conclusions

Among children and adolescents 6–17 years old, similar foods ranked among those frequently consumed. However, frequency-differentiated intake patterns exist for beverages and snack foods by food security across age groups. The main findings reported in this paper may inform dietary recommendations, development of population-specific dietary assessment tools, interventions, menus, and the composition of food packages, and food policy for food insecure children, and adolescents.

## Figures and Tables

**Table 1:** Characteristics of food secure, low and very low food secure U.S. children and adolescents ages 6–17 years using the National Health and Nutrition Examination Survey 2007–2014 ^a^.

	6–17 Years		6–11 Years (*n* = 4437)							12–17 Years (*n* = 3686)						
			Food Secure		Low Food Secure		Very Low Food Secure			Food Secure		Low Food Secure		Very Low Food Secure		
Characteristic	*n*	%	*n*	%	*n*	%	*n*	%	χ^2^ *p*-value ^b^	*n*	%	*n*	%	*n*	%	χ^2^ *p*-value ^b^
Total	8123	100	3854	90	510	9	73	1		3178	89	426	10	82	2	
Sex									0.32							0.15
Male	4152	50	1941	51	272	54	40	57		1625	48	233	54	41	39	
Female	3971	50	1913	49	238	46	33	43		1553	52	193	46	41	61	
Survey Year									0.48							0.64
2007–2008	1990	25	939	24	147	25	24	30		738	25	120	31	22	24	
2009–2010	2106	25	1024	26	105	18	15	18		829	25	115	25	18	21	
2011–2012	2011	25	986	25	139	30	19	23		759	25	94	26	14	24	
2013–2014	2016	25	905	25	119	26	15	29		852	25	97	18	28	31	
Poverty-Income-Ratio									<0.0001 *^,c^							<0.0001 *^,c^
0.00–0.99	2504	24	1142	23	258	49	48	69		797	18	215	48	44	58	
1.00–1.99	2076	24	948	22	187	40	18	31		766	22	134	37	23	34	
2.00–2.99	1029	16	511	17	41	8	0	0		437	16	36	8	4	8	
3.00–5.00	1977	37	1016	39	12	3	0	0		933	43	16	7	0	0	
Race/Ethnicity									<0.0001 *							0.0004
Mexican American and Other Hispanic	2873	22	1325	21	221	34	41	54		1063	19	180	30	43	44	
Non-Hispanic White	2289	56	1138	57	111	38	9	19		919	60	94	39	18	40	
Non-Hispanic Black	2079	14	983	13	138	21	19	22		805	14	119	21	15	13	
Other-Race including Multi-Racial	882	8	408	8	40	7	4	4		391	7	33	10	6	3	
Body Mass Index Status ^d^									0.001 *							0.12
Underweight	280	3	126	4	23	5	1	1		110	3	16	5	4	5	
Normal weight	4819	61	2366	62	264	49	37	52		1877	63	230	54	45	50	
Overweight	1324	16	599	16	99	22	15	18		523	15	69	14	19	26	
Obese	1700	19	763	18	124	24	20	28		668	19	111	27	14	19	

^a^ Total numbers do not always add to sample size due to missing values. Percents do not always add to 100 due to rounding. Estimate represents weighted percent. ^b^ Rao Scott F adjusted χ2 *p*-value is shown, statistical significance for differences among food secure, low food secure, and very low food secure among each respective age groups is indicated when *p* ≤ 0.02 using a Bonferroni type adjustment for multiple comparisons indicated by “*”. Sample weights were appropriately constructed and applied to this analysis as directed by National Center for Health Statistics. Weights were rescaled so that the sum to the weights matched the survey population at the midpoint of the 8 years, 2007–2014. ^c^ Because of one or more empty cells, food secure and very low food secure were collapsed in order to compute Rao Scott F adjusted χ2 *p*-value. ^d^ Body Mass Index status was classified based on Centers for Disease Control and Prevention values as per: https://www.cdc.gov/nccdphp/dnpao/growthcharts/resources/sas.htm; <5% (underweight), 5 ≥ 85% (normal weight), 85 ≥ 95% (overweight), ≥95% (obese).

**Table 2:** What We Eat in America broad food category ^a^ intake comparisons by frequency and energy among food secure and insecure (low and very low food secure) U.S. children and adolescents 6–11 and 12–17 years using the National Health and Nutrition Examination Survey 2007–2014 ^b^.

	6–11 Years (*n* = 4437)											
	Food Secure		Food Insecure		χ^2^ *p*-Value		Low Food Secure		Very Low Food Secure		χ^2^ *p*-Value	
WWEIA Broad Food Category ^a,c^	Wtd % ^d^ of Reported Foods (SE)	Wtd % ^d^ of Reported Energy (SE)	Wtd % ^d^ of Reported Foods (SE)	Wtd % ^d^ of Reported Energy (SE)	Foods ^e^	Energy ^e^	Wtd % ^d^ of Reported Foods (SE)	Wtd % ^d^ of Reported Energy (SE)	Wtd % ^d^ of Reported Foods (SE)	Wtd % ^d^ of Reported Energy (SE)	Foods ^f^	Energy ^f^
Milk/Dairy ^g^	12.6 (0.2)	11.6 (0.3)	12.0 (0.6)	11.0 (0.5)	0.31	0.36	12.0 (0.6)	10.8 (0.6)	11.7 (0.8)	12.7 (1.2)	0.47	0.34
Protein ^h^	9.3 (0.2)	12.1 (0.3)	9.6 (0.5)	11.6 (0.7)	0.59	0.57	9.3 (0.5)	11.1 (0.9)	12.0 (0.8)	15.4 (1.0)	0.11	0.14
Mixed Dish ^i^	8.0 (0.2)	22.1 (0.6)	9.3 (0.6)	24.8 (1.9)	0.04 *	0.19	9.4 (0.7)	25.3 (2.1)	8.9 (0.9)	20.2 (1.7)	0.05	0.15
Grain ^j^	11.2 (0.2)	14.3 (0.3)	11.4 (0.4)	13.7 (0.8)	0.72	0.49	11.4 (0.5)	13.8 (0.8)	10.9 (0.8)	12.8 (1.1)	0.84	0.62
Snack/Sweet ^k^	16.1 (0.3)	21.0 (0.5)	14.4 (0.6)	18.3 (0.9)	0.02*	0.02 *	14.5 (0.6)	18.1 (0.9)	13.9 (1.7)	20.1 (1.8)	0.05	0.02 *
Fruit ^l^	5.8 (0.2)	2.8 (0.1)	5.1 (0.4)	2.7 (0.3)	0.17	0.67	4.8 (0.4)	2.5 (0.3)	7.4 (1.1)	4.0 (0.5)	0.07	0.24
Vegetable ^m^	6.5 (0.2)	3.6 (0.2)	6.5 (0.7)	4.3 (0.5)	0.98	0.26	6.4 (0.7)	4.4 (0.6)	7.8 (0.9)	3.3 (0.5)	0.72	0.27
Beverage ^n^	11.4 (0.2)	8.9 (0.2)	12.7 (0.6)	9.9 (0.6)	0.02 *	0.11	12.8 (0.6)	9.9 (0.7)	12.0 (1.1)	9.8 (1.3)	0.04	0.22
Water ^o^	9.0 (0.2)	0.0 (0.0)	8.9 (0.6)	0.0 (0.0)	0.90	0.18	9.0 (0.6)	0.0 (0.0)	8.4 (0.8)	0.0 (0.0)	0.92	0.18
Fat/Oil ^p^	3.3 (0.1)	1.5 (0.1)	3.1 (0.3)	1.7 (0.2)	0.56	0.55	3.3 (0.3)	1.8 (0.3)	1.3 (0.2)	0.8 (0.1)	0.03	0.21
Cond ^q^/Sauce ^r^	4.2 (0.2)	0.7 (0.0)	4.6 (0.4)	0.8 (0.2)	0.44	0.57	4.7 (0.4)	0.9 (0.2)	3.7 (1.0)	0.5 (0.2)	0.48	0.54
Sugars ^s^	2.4 (0.1)	1.1 (0.1)	2.2 (0.2)	1.1 (0.2)	0.46	0.88	2.2 (0.2)	1.2 (0.2)	1.9 (0.6)	0.3 (0.1)	0.70	0.12
	**12–17 Years (*n* = 3686)**											
	**Food Secure**		**Food Insecure**		**χ^2^*p*-Value**		**Low Food Secure**		**Very Low Food Secure**		**χ^2^*p*-Value**	
**WWEIA Broad Food Category ^a,c^**	**Wtd % ^d^ of Reported Foods (SE)**	**Wtd % ^d^ of Reported Energy (SE)**	**Wtd % ^d^ of Reported Foods (SE)**	**Wtd % ^d^ of Reported Energy (SE)**	**Foods ^e^**	**Energy ^e^**	**Wtd % ^d^ of Reported Foods (SE)**	**Wtd % ^d^ of Reported Energy (SE)**	**Wtd % ^d^ of Reported Foods (SE)**	**Wtd % ^d^ of Reported Energy (SE)**	**Foods ^f^**	**Energy ^f^**
Milk/Dairy ^g^	10.7 (0.3)	9.4 (0.3)	9.8 (0.4)	8.3 (0.5)	0.06	0.08	9.7 (0.4)	8.1 (0.6)	10.3 (0.5)	9.5 (0.8)	0.17	0.20
Protein ^h^	9.6 (0.3)	12.8 (0.6)	9.8 (0.5)	12.7 (0.9)	0.77	0.98	9.7 (0.5)	12.2 (0.9)	10.6 (0.5)	16.2 (0.8)	0.74	0.35
Mixed Dish ^i^	8.8 0.2)	24.6 (0.7)	10.1 (0.7)	25.3 (1.5)	0.09	0.66	10.3 (0.8)	25.8 (1.6)	8.7 (0.3)	21.9 (0.9)	0.08	0.55
Grain ^j^	10.6 (0.2)	12.8 (0.3)	10.8 (0.4)	12.7 (0.7)	0.61	0.84	10.9 (0.4)	12.9 (0.7)	10.5 (0.8)	11.3 (1.0)	0.87	0.71
Snack/Sweet ^k^	14.3 (0.4)	18.7 (0.6)	13.3 (0.6)	18.6 (1.3)	0.18	0.96	13.5 (0.6)	18.7 (1.3)	12.4 (0.5)	18.5 (0.9)	0.28	0.99
Fruit ^l^	4.3 (0.3)	2.0 (0.1)	4.1 (0.4)	2.0 (0.3)	0.66	0.88	4.1 (0.5)	2.0 (0.2)	4.1 (0.5)	2.3 (0.3)	0.92	0.92
Vegetable ^m^	7.9 (0.3)	4.2 (0.2)	7.9 (0.6)	3.9 (0.4)	0.96	0.49	7.6 (0.6)	3.8 (0.5)	9.8 (0.7)	4.3 (0.3)	0.63	0.69
Beverage ^n^	12.7 (0.2)	11.2 (0.3)	15.4 (0.6)	13.0 (0.7)	<0.0001 *	0.03 *	15.5 (0.5)	13.1 (0.7)	14.4 (0.7)	12.2 (0.7)	0.0001 *	0.05
Water ^o^	10.3 (0.3)	0.1 (0.0)	8.7 (0.5)	0.1 (0.0)	0.004 *	0.77	8.6 (0.6)	0.1 (0.1)	8.8 (0.5)	0.1 (0.1)	0.004 *	0.92
Fat/Oil ^p^	3.6 (0.2)	1.9 (0.2)	3.5 (0.3)	1.6 (0.2)	0.90	0.18	3.4 (0.3)	1.4 (0.2)	4.0 (0.6)	2.6 (0.2)	0.83	0.10
Cond ^q^/Sauce ^r^	5.0 (0.2)	0.9 (0.1)	4.5 (0.5)	0.7 (0.1)	0.32	0.37	4.4 (0.5)	0.8 (0.1)	4.7 (0.4)	0.6 (0.1)	0.60	0.59
Sugars ^s^	2.0 (0.1)	1.0 (0.1)	1.8 (0.2)	0.7 (0.1)	0.68	0.08	2.0 (0.3)	0.8 (0.1)	1.4 (0.2)	0.5 (0.1)	0.57	0.06

^a^ The What We Eat in America (WWEIA) broad food categories were applied to categorize all foods and beverages reported in a single day to 14 broad food groups. ^b^ Survey weights and adjustments for the complex survey design were applied to represent the non-institutionalized U.S. population. Total numbers and percentages do not always add up to sample size due to missing values and rounding. ^c^ “Alcohol” and “Other” WWEIA category removed because of <20 reports. ^d^ Wtd % stands for the estimated weighted percent of all reports of foods or beverages or energy from reported foods or beverages reported in a single day that are included in a food group; SE = Standard Error ^e^ Statistical significance at *p* ≤ 0.05 for comparison of food secure vs. food insecure using the Rao–Scott modified chi-square statistic ^f^ Statistical significance at *p* ≤ 0.02 using a Bonferroni type adjustment for multiple comparisons indicated by “*”for comparison of food secure vs. low food secure vs. very low food secure and using the Rao–Scott modified chi-square statistic. ^g^ Milk, flavored milk, dairy drinks and substitutes, cheese and yogurt. ^h^ Meats, poultry, seafood, eggs, cured meats/poultry, and plant-based protein foods. ^i^ Mixed dishes containing meat, poultry seafood; grain-based; Asian; Mexican; pizza; sandwiches, and soups. ^j^ Cooked grains, breads, rolls, tortillas, quick breads, and bread products, ready-to-eat cereals, and cooked cereals. ^k^ Savory snacks, crackers, snack/meal bars, sweet bakery products, candy and other desserts. ^l^ Fresh fruits, dried fruits, and fruit salads. ^m^ Vegetables and white potatoes. ^n^ 100% juice, diet beverages, sweetened beverages, coffee and tea. ^o^ Plain water and flavored or enhanced water. ^p^ Butter and animal fats, margarine, cream cheeses, cream, mayonnaise, salad dressings and vegetable oils. ^q^ Condiment. ^r^ Tomato-based, soy-based, mustard, olives, pickled vegetables, pasta sauces, dips, gravies, and other sauces. ^s^ Sugars, honey, jams, syrups, and toppings.

**Table 3:** Top 25 most frequently consumed What We Eat in America food or beverage sub-categories ^a^, unweighted frequency of reported intake from food or beverage sub-categories, weighted percent and standard error of weighted percent of reported food or beverage sub-categories among all reported sub-categories for food secure, low, and very low food secure U.S. children and adolescents aged 6–11 and 12–17 years using the National Health and Nutrition Examination Survey 2007–2014.

	6–11 Years (*n* = 4437)									12–17 Years (*n* = 3686)								
	Food Secure			Low Food Secure			Very Low Food Secure			Food Secure			Low Food Secure			Very Low Food Secure		
Rank	WWEIA Sub-Category ^a^	Freq ^b^	Wtd % ^c,d^ (SE)	WWEIA Sub-Category ^a^	Freq ^b^	Wtd % ^c,d^ (SE)	WWEIA Sub-Category ^a^	Freq ^b^	Wtd % ^c,d^ (SE)	WWEIA Sub-Category ^a^	Freq ^b^	Wtd % ^c,d^ (SE)	WWEIA Sub-Category ^a^	Freq ^b^	Wtd % ^c,d^ (SE)	WWEIA Sub-Category ^a^	Freq ^b^	Wtd % ^c,d^ (SE)
Total		58,077	100		7322	100		1146	100		41,404	100		5187	100		981	100
1	Tap water	3082	5.9 (0.3)	Tap water	350	4.8 (0.4)	Reduced fat milk	53	4.6 (0.4)	Tap water	2166	5.9 (0.3)	Soft drinks	306	5.6 (0.5)	Bottled water	50	5.2 (0.6)
2	Fruit drinks	2247	3.2 (0.1)	Fruit drinks	300	4.3 (0.5)	Tap water	51	4.3 (0.4)	Soft drinks	1957	4.3 (0.2)	Tap water	226	4.5 (0.5)	Soft drinks	63	5.1 (0.4)
3	Yeast breads	1687	3.1 (0.1)	Bottled water	304	4.1 (0.6)	Fruit drinks	46	4.1 (0.9)	Bottled water	1841	4.1 (0.2)	Bottled water	218	4.0 (0.5)	Cheese	35	4.4 (0.5)
4	Cheese	1629	3.1 (0.1)	Soft drinks	296	3.9 (0.5)	Bottled water	41	4.0 (0.6)	Cheese	1295	3.4 (0.2)	Yeast breads	155	3.2 (0.3)	Tea	19	3.6 (0.4)
5	Soft drinks	1925	3.0 (0.2)	Yeast breads	212	3.2 (0.3)	Soft drinks	38	3.4 (0.7)	Yeast breads	1193	2.9 (0.1)	Fruit drinks	179	3.0 (0.4)	Tap water	30	3.5 (0.2)
6	Reduced Fat Milk	1681	2.9 (0.2)	Tomato-based condiments	244	3.0 (0.3)	Apples	24	2.2 (0.4)	Tomato-based condiments	1215	2.8 (0.2)	Reduced fat milk	140	3.0 (0.4)	Yeast breads	36	3.3 (0.5)
7	Bottled water	1989	2.9 (0.2)	Cheese	186	2.8 (0.4)	Candy w/o chocolate	22	2.2 (0.8)	Reduced fat milk	1013	2.8 (0.2)	Cheese	145	2.8 (0.3)	Chicken, whole pieces	24	2.8 (0.2)
8	Tomato-based condiments	1523	2.6 (0.1)	Reduced fat milk	200	2.6 (0.3)	Cheese	25	2.2 (0.4)	Fruit drinks	1,189	2.4 (0.1)	Pizza	123	2.2 (0.3)	Fruit drinks	32	2.7 (0.5)
9	Candy w/o chocolate	1415	2.6 (0.2)	Ready-to-eat cereal, higher sugar (21.2g/100g)	180	2.3 (0.3)	Rolls and buns	19	2.1 (0.6)	Cookies and brownies	945	2.1 (0.1)	Tomato-based condiments	146	2.1 (0.3)	Lettuce and lettuce salads	20	2.6 (0.2)
10	Cookies and brownies	1398	2.6 (0.1)	Cookies and brownies	153	1.9 (0.2)	Ready-to-eat cereal, higher sugar (21.2g/100g)	30	2.1 (0.4)	Rolls and buns	725	1.9 (0.1)	Cookies and brownies	112	2.0 (0.2)	Cold cuts and cured meats	24	2.5 (0.5)
11	Ready-to-eat cereal, higher sugar (21.2g/100g)	1333	2.2 (0.1)	Jams, syrups, toppings	112	1.8 (0.2)	Candy w/chocolate	15	2.1 (0.3)	Pizza	856	1.9 (0.1)	Tea	96	2.0 (0.3)	Candy w/chocolate	22	2.4 (0.3)
12	Jams, syrups, toppings	1005	1.8 (0.1)	French fries and fried white potatoes	130	1.8 (0.2)	Pizza	26	2.0 (0.2)	Ready-to-eat cereal, higher sugar (21.2g/100g)	776	1.8 (0.1)	Corn tortilla and other chips	108	2.0 (0.2)	Tomato-based condiments	24	2.4 (0.2)
13	Pizza	1117	1.8 (0.1)	Cold cuts and cured meats	101	1.7 (0.2)	Yeast breads	25	2.0 (0.3)	Candy w/o chocolate	821	1.8 (0.1)	Ice cream and frozen dairy desserts	82	1.8 (0.3)	Rolls and buns	12	2.2 (0.2)
14	Corn tortilla and other chips	978	1.6 (0.1)	Whole milk	127	1.7 (0.3)	Tomato-based condiments	26	1.9 (0.5)	Corn tortilla and other chips	827	1.7 (0.1)	Ready-to-eat cereal, higher sugar (21.2g/100g)	99	1.8 (0.3)	Candy w/o chocolate	19	2.0 (0.1)
15	Rolls and buns	873	1.6 (0.1)	Corn tortilla and other chips	124	1.7 (0.2)	Eggs and omelets	22	1.9 (0.4)	Cold cuts and cured meats	680	1.6 (0.1)	Citrus juice	69	1.6 (0.3)	Reduced fat milk	31	1.9 (0.4)
16	French fries and fried white potatoes	931	1.5 (0.1)	Pizza	147	1.6 (0.2)	Chicken, whole pieces	18	1.9 (0.4)	Tea	589	1.6 (0.1)	Chicken, whole pieces	97	1.6 (0.2)	Ready-to-eat cereal, higher sugar (21.2g/100g)	19	1.8 (0.2)
17	Ice cream and frozen dairy desserts	861	1.5 (0.1)	Candy w/o chocolate	143	1.6 (0.2)	Ice cream and frozen dairy desserts	21	1.9 (0.3)	French fries and fried white potatoes	678	1.5 (0.1)	Lettuce and lettuce salads	77	1.5 (0.2)	Whole milk	12	1.7 (0.1)
18	Apples	838	1.5 (0.1)	Ice cream and frozen dairy desserts	93	1.6 (0.2)	Citrus juice	18	1.6 (0.3)	Chicken, whole pieces	706	1.4 (0.1)	Rolls and buns	95	1.5 (0.2)	Mustard and other condiments	14	1.7 (0.2)
19	Cold cuts and cured meats	775	1.4 (0.1)	Apples	99	1.5 (0.2)	Reduced fat flavored milk	12	1.6 (0.3)	Lettuce and lettuce salads	595	1.4 (0.1)	French fries and fried white potatoes	100	1.5 (0.2)	Bananas	13	1.7 (0.2)
20	Whole milk	911	1.3 (0.1)	Crackers, excludes saltines	62	1.5 (0.3)	Rice	15	1.6 (0.4)	Jams, syrups, toppings	486	1.3 (0.1)	Cold cuts and cured meats	76	1.5 (0.2)	Corn tortilla and other chips	15	1.7 (0.2)
21	Lowfat milk	551	1.3 (0.1)	Chicken, whole pieces	93	1.3 (0.2)	Lettuce and lettuce salads	17	1.6 (0.2)	Ice cream and frozen dairy desserts	501	1.2 (0.1)	Candy w/o chocolate	96	1.5 (0.2)	French fries and fried white potatoes	13	1.6 (0.1)
22	Nuts and seeds	560	1.2 (0.1)	Mayonnaise	65	1.2 (0.2)	Soups	21	1.5 (0.5)	Potato chips	523	1.2 (0.1)	Apples	57	1.3 (0.2)	Pizza	19	1.6 (0.2)
23	Chicken, whole pieces	800	1.2 (0.1)	Rolls and buns	99	1.2 (0.2)	Corn tortilla and other chips	25	1.5 (0.3)	Apples	485	1.2 (0.1)	Candy w/chocolate	60	1.3 (0.2)	Chicken patties, nuggets and tenders	9	1.5 (0.2)
24	Citrus juice	688	1.1 (0.1)	Citrus juice	111	1.1 (0.2)	Beans, peas, legumes	14	1.5 (0.2)	Eggs and omelets	488	1.0 (0.1)	Soups	49	1.2 (0.4)	Doughnuts, sweet rolls, pastries	11	1.5 (0.1)
25	Potato chips	661	1.1 (0.1)	Potato chips	81	1.1 (0.2)	Cookies and brownies	20	1.5 (0.2)	Crackers, excludes saltines	286	1.0 (0.1)	Sugars and honey	46	1.2 (0.2)	Cookies and brownies	21	1.3 (0.2)

^a^ The What We Eat in America Food Sub-Categories were applied to categorize all foods and beverages to 150 unique sub-categories. ^b^ The frequency that a food or beverages sub-category was reported without dietary weights. ^c^ Survey weights and adjustments for the complex survey design were applied to represent the non-institutionalized U.S. population. ^d^ Derived from the weighted frequency of the foods or beverages in a sub-category divided by the total weighted frequency of all foods or beverages (*n*) reported in all sub-categories in a single day, where *n* = 335,995,769 for food secure 6–11 years, *n* = 31,155,106 for low food secure 6–11 years, *n* = 4,126,641 for very low food secure 6–11 years, *n* = 290,965,789 for food secure 12–17 years, *n* = 28,444,012 for low food secure 12–17 years, *n* = 4,749,595 for very low food secure 12–17 years. Estimated weighted percent has been abbreviated by “Wtd %”; SE = Standard Error.

## Supplementary Materials

The following are available online at https://www.mdpi.com/2072-6643/12/2/304/s1, Table S1: Top 25 most frequently consumed foods or beverages, unweighted frequency of reported foods or beverages, weighted percent of reported foods or beverages, and standard error of weighted percent of reported foods or beverages among all reported foods or beverages for food secure, low, and very low food secure U.S. children and adolescents aged 6–11 and 12–17 years using the National Health and Nutrition Examination Survey 2007–2014.
