# Supplementary material for: Frequently Consumed Foods and Energy Contributions among Food Secure and Insecure U.S. Children and Adolescents
Source: Nutrients. 2020 Jan 23;12(2):304. doi: 10.3390/nu12020304 (PMC7070395; doi:10.3390/nu12020304)
Supplement: Supplementary file 1 [file nutrients-12-00304-s001.pdf]

Supplemental Table 1. Top 25 most frequently consumed foods or beverages, unweighted frequency of reported foods or beverages, weighted percent of reported foods or beverages, and standard error of weighted percent of reported foods or beverages among all reported foods or beverages for food secure, low, and very low food secure U.S. children and adolescents aged 6-11 and 12-17 years using the National Health and Nutrition Examination Survey 2007–2014.

	6-11 Years (*n* = 4,510)									12-17 Years (*n* = 3,686)								
	Food Secure			Low Food Secure			Very Low Food Secure			Food Secure			Low Food Secure			Very Low Food Secure		
**Rank**	**Food Code[a]**	**Freq[b]**	**Wtd % [c,d] (SE)**	**Food Code[a]**	**Freq[b]**	**Wtd % [c,d] (SE)**	**Food Code[a]**	**Freq[b]**	**Wtd % [c,d] (SE)**	**Food Code[a]**	**Freq[b]**	**Wtd % [c,d] (SE)**	**Food Code[a]**	**Freq[b]**	**Wtd % [c,d] (SE)**	**Food Code[a]**	**Freq[b]**	**Wtd % [c,d] (SE)**
Total		58,077	100		7,322	100		1,146	100		41,404	100		5,187	100		981	100
1	Tap Water	3,041	5.8 (0.3)	Tap Water	339	4.7 (0.4)	Tap Water	50	4.2 (0.4)	Tap Water	2,143	5.9 (0.3)	Tap Water	222	4.5 (0.5)	Unsweetened Bottled Water	50	5.2 (0.6)
2	Unsweetened Bottled Water	1,989	2.9 (0.2)	Unsweetened Bottled Water	304	4.1 (0.6)	Unsweetened Bottled Water	41	4.0 (0.6)	Unsweetened Bottled Water	1,841	4.1 (0.2)	Unsweetened Bottled Water	218	4.0 (0.5)	Tap Water	30	3.5 (0.2)
3	2% Cow's Milk	1,560	2.7 (0.2)	2% Cow's Milk	167	2.2 (0.3)	2% Cow's Milk	45	4.0 (0.4)	2% Cow's Milk	889	2.5 (0.2)	2% Cow's Milk	117	2.6 (0.4)	Raw Lettuce	18	2.5 (0.2)
4	Tomato Catsup	1,046	1.8 (0.1)	Tomato Catsup	154	1.9 (0.2)	Raw Apple	21	1.9 (0.3)	Tomato Catsup	680	1.6 (0.1)	Cola-Type Soft Drink	108	2.2 (0.3)	Cola-Type Soft Drink	29	2.5 (0.3)
5	Whole Cow's Milk	901	1.3 (0.1)	White Bread	100	1.6 (0.2)	Fruit Flavored Drink From Powder	11	1.6 (0.9)	Cola-Type Soft Drink	639	1.5 (0.1)	Fruit-Flavored, Caffeine Free Soft Drink	109	1.5 (0.2)	Whole Cow's Milk	12	1.7 (0.1)
6	1% Cow's Milk	550	1.3 (0.1)	Whole Cow's Milk	126	1.6 (0.3)	Fruit-Flavored, Caffeine Free Soft Drink	17	1.5 (0.3)	Raw Lettuce	542	1.3 (0.1)	Raw Lettuce	69	1.3 (0.2)	Raw Banana	13	1.7 (0.2)
7	Raw Apple	695	1.2 (0.1)	Fruit-Flavored, Caffeine Free Soft Drink	121	1.5 (0.2)	Cola-Type Soft Drink	16	1.5 (0.4)	Soft White Roll	422	1.2 (0.1)	Raw Apple	54	1.3 (0.2)	2% Cow's Milk	25	1.7 (0.3)
8	Fruit-Flavored,	741	1.0 (0.1)	Cola-Type Soft Drink	91	1.3 (0.3)	Raw Lettuce	15	1.5 (0.2)	Fruit-Flavored,	645	1.0 (0.1)	Tomato Catsup	81	1.2 (0.2)	Soft White Roll	9	1.6 (0.2)

	Caffeine Free Soft Drink									Caffeine Free Soft Drink								
8	Soft White Roll	568	1.0 (0.1)	Raw Apple	82	1.2 (0.2)	Raw Banana	14	1.4 (0.3)	Raw Apple	427	1.0 (0.1)	White Bread	70	1.2 (0.2)	Brewed, Pre-sugar-sweetened Black Iced Tea	7	1.6 (0.1)
9	Cola-Type Soft Drink	613	0.9 (0.1)	1% Cow's Milk	68	1.1 (0.2)	Soft White Roll	12	1.4 (0.5)	1% Cow's Milk	386	1.0 (0.1)	White Sugar	41	1.0 (0.2)	Prepackaged or Deli Sliced Ham	9	1.4 (0.1)
10	White Bread	564	0.9 (0.1)	White Potato French Fries	78	1.0 (0.1)	White Bread	15	1.4 (0.2)	White Bread	421	0.9 (0.1)	Orange Juice	47	0.9 (0.2)	Fast Food White Potato French Fries	8	1.4 (0.1)
11	Apple Juice	525	0.8 (0.1)	Reduced Sugar Fruit Juice Drink	40	1.0 (0.2)	Orange Juice w/Calcium	14	1.3 (0.2)	Skim Cow's Milk	226	0.9 (0.1)	Soft White Roll	51	0.9 (0.2)	Raw Tomatoes	14	1.3 (0.2)
12	Skim Cow's Milk	256	0.8 (0.1)	Apple Juice	77	0.9 (0.2)	Whole Cow's Milk	20	1.3 (0.2)	Whole Cow's Milk	404	0.9 (0.1)	Fruit Flavored Drink From Powder	57	0.9 (0.2)	White Sugar	9	1.2 (0.2)
13	White Potato French Fries	534	0.8 (0.1)	Corn Tortilla Chips	60	0.8 (0.2)	Raw Orange	10	1.1 (0.4)	Corn Tortilla Chips	379	0.9 (0.1)	Whole Cow's Milk	56	0.9 (0.2)	White Bread	12	1.0 (0.1)
14	Raw Lettuce	458	0.8 (0.1)	Soft White Roll	62	0.8 (0.2)	Tomato Catsup	14	1.1 (0.2)	Fruit Flavored Soft Drink	245	0.8 (0.1)	Wheat Bread	33	0.8 (0.2)	Mayonnaise	14	1.0 (0.2)
15	Corn Tortilla Chips	442	0.7 (0.1)	Mayonnaise	39	0.8 (0.2)	Peanut Butter	11	1.0 (0.2)	Raw Tomatoes	317	0.8 (0.1)	Fruit-Flavored Soft Drink	34	0.8 (0.3)	Raw Apple	10	1.0 (0.3)
16	Peanut Butter	334	0.7 (0.1)	Fruit Flavored Drink From Powder	64	0.8 (0.2)	Ice Cream	9	1.0 (0.2)	White Potato French Fries	376	0.8 (0.1)	White Potato French Fries	56	0.8 (0.1)	Ground Beef or Patty	6	1.0 (0.1)

17	Hard Candy	418	0.7 (0.1)	American Cheese	45	0.8 (0.3)	White Sugar	10	1.0 (0.4)	Red, Cooked Salsa	312	0.7 (0.1)	Raw Tomatoes	37	0.8 (0.2)	Tomato Catsup	11	1.0 (0.1)
18	Fruit Juice Drink	504	0.7 (0.0)	Fruit Juice Drink	55	0.7 (0.1)	2% Chocolate Milk	5	0.9 (0.3)	Chocolate Chip Cookie	282	0.6 (0.1)	Corn Puffs or Twists	35	0.7 (0.2)	Creamy Dressing	5	1.0 (0.3)
19	Raw Grapes	316	0.6 (0.1)	Peanut Butter	41	0.7 (0.2)	Raw Carrots	7	0.8 (0.2)	American Cheese	243	0.6 (0.1)	American Cheese	29	0.6 (0.1)	Breaded Chicken Patty Fillet or Tenders	5	1.0 (0.1)
20	Chocolate Chip Cookie	305	0.6 (0.1)	Orange Juice	51	0.6 (0.1)	Corn Tortilla	11	0.8 (0.1)	Raw Banana	238	0.6 (0.1)	Mustard	29	0.6 (0.1)	Mustard	5	0.9 (0.1)
21	Raw Bananna	398	0.6 (0.0)	Raw Lettuce	48	0.6 (0.1)	Ready-To-Eat Cereal	9	0.8 (0.3)	Ice Cream	227	0.6 (0.1)	Chocolate Chip Cookie	35	0.6 (0.1)	Red, Cooked Salsa	8	0.8 (0.1)
22	Ice Cream	325	0.6 (0.0)	Ground Beef or Patty	31	0.6 (0.2)	Red Cooked Salsa	9	0.8 (0.3)	Orange Juice	304	0.5 (0.1)	Prepackaged or Deli Sliced Ham	37	0.6 (0.1)	M&M's Milk Chocolate Candies	2	0.8 (0.1)
23	Reduced Sugar Fruit Juice Drink	376	0.6 (0.1)	Snack Cracker	22	0.6 (0.2)	Apple Juice	7	0.7 (0.3)	Ground Beef or Patty	220	0.5 (0.1)	Corn Tortilla Chips	38	0.6 (0.1)	Fruit Juice Drink	6	0.8 (0.1)
24	Fruit Flavored Drink From Powder	373	0.6 (0.1)	2% Chocolate Milk	40	0.6 (0.2)	Raw Tomatoes	10	0.7 (0.2)	Peanut Butter	161	0.5 (0.1)	Mayonnaise	29	0.6 (0.1)	American Cheese	5	0.8 (0.2)
25	Orange Juice	347	0.5 (0.1)	Ice Cream	30	0.5 (0.1)	Thin Crust Cheese Pizza	5	0.7 (0.2)	Apple Juice	240	0.5 (0.1)	Orange Juice w/Calcium	19	0.6 (0.2)	Fruit-Flavored Soft Drink	10	0.8 (0.2)

[a]U.S. Department of Agriculture Long Food Code Description, order of words may be rearranged to improve readability [b] The frequency that a food or beverages was reported without dietary weights. [c] Survey weights and adjustments for the complex survey design were applied to represent the non-institutionalized U.S. population. [d] Derived from the estimated weighted frequency of the individual food or beverage divided by the total weighted frequency of all foods and beverages *(n)* reported in a single day, where n = 335,995,769 for food secure 6–11 years, n = 31,155,106 for low food secure 6–11 years, n = 4,126,641 for very low food secure 6–11 years, n = 290,965,789 for food secure 12–17 years, n = 28,444,012 for low food secure 12–17 years, n = 4,749,595 for very low food secure 12–17 years. Estimated weighted percent has been abbreviated by "Wtd %"; SE=Standard Error.
